# Parents’ Report of Canadian Elementary School Children’s Physical Activity and Screen Time during the COVID-19 Pandemic: A Longitudinal Study

**DOI:** 10.3390/ijerph182312352

**Published:** 2021-11-24

**Authors:** Emma Ostermeier, Patricia Tucker, Andrew Clark, Jamie A. Seabrook, Jason Gilliland

**Affiliations:** 1 Health and Rehabilitation Sciences, Faculty of Health Sciences, The University of Western Ontario, London, ON N6A 3K7, Canada; eosterme@uwo.ca; 2 Child Health and Physical Activity Laboratory, School of Occupational Therapy, The University of Western Ontario, London, ON N6A 3K7, Canada; ttucker2@uwo.ca; 3 Human Environments Analysis Laboratory, Department of Geography and Environment, The University of Western Ontario, London, ON N6A 3K7, Canada; aclark2@uwo.ca (A.C.); jseabro2@uwo.ca (J.A.S.); 4 School of Occupational Therapy, Faculty of Health Sciences, The University of Western Ontario, London, ON N6A 3K7, Canada; 5 Children’s Health Research Institute, London, ON N6C 2V5, Canada; 6 School of Food and Nutritional Sciences, Brescia University College, London, ON N6G 1H2, Canada; 7 Department of Epidemiology & Biostatistics, The University of Western Ontario, London, ON N6A 3K7, Canada; 8 Department of Paediatrics, The University of Western Ontario, London, ON N6A 3K7, Canada; 9 Lawson Health Research Institute, London, ON N6A 4V2, Canada; 10 Department of Geography and Environment, The University of Western Ontario, London, ON N6A 3K7, Canada; 11 School of Health Studies, The University of Western Ontario, London, ON N6A 3K7, Canada

**Keywords:** physical activity, screen time, child, parent, coronavirus, public health, protocols, Canada

## Abstract

COVID-19 public health protocols have altered children’s daily routines, limiting their physical activity opportunities. The purpose of this study was to examine how the COVID-19 pandemic affected children’s (ages 10–12 years) physical activity and screen time, and to explore the impact of gender, socioeconomic status (SES), and public health constraints (i.e., facility use and social interaction) on the changes in children’s health behaviors. Online surveys were disseminated to parents at two time points: before COVID-19 (May 2019 to February 2020) and during COVID-19 (November to December 2020). Wilcoxon signed-rank tests were used to assess changes in physical activity and screen time, and for subgroup analyses. Parents (*n* = 95) reported declines in children’s physical activity (Z = −2.53, *p* = 0.01, *d* = 0.18), and increases in weekday (Z = −4.61, *p* < 0.01, *d* = 0.33) and weekend screen time (Z = −3.79, *p* < 0.01, *d* = 0.27). Significant changes in physical activity and screen time behaviors were identified between gender, SES, and facility use groups. All social interaction groups underwent significant changes in screen time. Overall, COVID-19 protocols have negatively influenced children’s physical activity and screen time. Due to the negative consequences of inactivity and excessive screen time, resources must be made available to support families during the pandemic.

## 1. Introduction

In March 2020, The World Health Organization (WHO) declared the SARS-Cov-2 (COVID-19) virus a global pandemic due to the transmission rate and the severity of the disease [1]. To protect citizens, policies and regulations, such as physical distancing, limited gathering sizes, and stay-at-home orders, have been enacted [2]. While these protocols protect the community against viral transmission, the introduction of restrictive public health protocols has substantially altered children’s everyday activities. For example, the mandated closure of schools and community centers and gathering restrictions have resulted in diminished interactions with friends and the termination of many after-school activities [2]. As children are currently experiencing physical and social barriers to their usual activities, there are concerns about how the COVID-19 protocols may have exacerbated the already problematic physical activity and screen time behaviors among children [3].

Prior to COVID-19, physical inactivity and excessive screen time have been a priority area for WHO and government agencies due to the well-known negative impacts on children’s health and well-being, including their cardiometabolic health, bone mineral density, motor skill development, anxiety, depression, academic achievement, and cognitive functioning [4,5,6,7,8,9]. WHO’s guidelines for physical activity and sedentary behaviors recommend that children accumulate an average of 60 min of moderate-to-vigorous physical activity (MVPA) per day and limit screen-based activities [4], and many countries have adopted these recommendations into their national movement guidelines. In Canada, the responses to the 2016–2017 Canadian Health Measures Survey reveal that 61% of Canadian children (ages 5 to 17 years) were not meeting the 60 min of daily MVPA [10]. Likewise, 72% of children surpassed the 2 h maximum screen time recommendation in the Canadian 24-Hour Movement Guidelines for Children and Youth [11], with greater amounts of screen time reported on weekends compared to weekdays [12]. There are various factors associated with children’s movement behaviors. For instance, research consistently demonstrates that girls are less active than boys [11], while boys are less likely to meet the screen time recommendations compared to girls [13]. Physical activity and screen time disparities are also correlated with socioeconomic status (SES). Specifically, children from lower-income households tend to engage in fewer minutes of physical activity and more minutes of screen time compared to children that live in higher-income households [10,14].

COVID-19 protocols have made partaking in physical activity more difficult for children, with recreational facilities, park/playgrounds, and organized activities restricted or unavailable [15]. In addition to the government protocols, parents may restrict children’s use of public places due to transmission concerns, or they may question their children’s ability to maintain physical distancing during activities and may opt for their family to only socialize within their ‘bubble’ (i.e., family members and/or friends that are isolating together). Since children are no longer able to attend their usual after-school activities, screen-based activities may act as a primary replacement [16]. To our knowledge, this is the first longitudinal study that utilizes baseline physical activity and screen time data collected prior to the pandemic to explore the impact of COVID-19 on Canadian children’s physical activity levels and screen time. The primary purpose of this study was to examine how the COVID-19 pandemic impacted children’s physical activity and screen time. The secondary objective was to explore differences in children’s physical activity and screen time based on gender, SES and COVID-19 constraints (i.e., facility use and social interaction).

## 2. Materials and Methods

### 2.1. Study Design and Participants

As part of the Grade 5 ACT-i-Pass (G5AP) study [17], parents of grade six children (ages 10 to 12 years) were recruited to complete online surveys about their children’s physical activity and screen time. Parents who consented to receive updates about the G5AP were contacted to participate in the study via the email address provided on the G5AP registration form. Using a longitudinal design, surveys were disseminated to parents at two time points: before COVID-19 (May 2019 to February 2020) and during COVID-19 (November to December 2020); when London, Ontario was in the orange ‘restrict’ level of COVID-19 public health measures. At this time, children had access to many indoor (e.g., community centers) and outdoor (e.g., parks and playgrounds) recreational places with restrictions, including shorter activity times, reduced facility capacity, and limited activity options [18]. Participants had to meet the following inclusion criteria: (1) parent of a grade six child (ages 10–12 years) that was enrolled in the G5AP; and (2) lived or attended a school in London, Ontario. Parents were recruited to complete the before COVID-19 baseline survey in the 2019–2020 school year following the completion of the G5AP registration form. The before COVID-19 survey was regularly available to parents prior to the development of this study. The COVID-19 survey was disseminated in November and December 2020 to past G5AP parents who consented to receive program updates via email. Participant data were included in the analysis if parents completed both the before COVID-19 and COVID-19 surveys. 

### 2.2. Measures

#### 2.2.1. Physical Activity and Screen Time

The surveys utilized the physical activity and screen time questions from the Canadian Health Measures Survey, a validated tool for measuring health and movement behaviors [19,20]. The questions focused on children’s physical activity behaviors (e.g., weekly activity levels and activity preferences), screen time (e.g., daily screen viewing), and demographics (e.g., gender, age, ethnicity, and family organization). The COVID-19 survey also integrated questions about the families’ COVID-19 constraints, including their child’s school delivery model, use of recreational facilities, and interactions with friends and family members. 

To measure physical activity, parents were asked how many days in the past week their child took part in 60-min of physical activity. As the Canadian movement guidelines emphasize the importance of MVPA, physical activity was defined as any activity that makes their child out of breath or increases their body temperature. For screen time, parents were asked to report how many hours per day their child used a screen-based device (e.g., computer, TV, or mobile device) for activities other than schoolwork. As previous studies have shown that screen time on school days and weekends differ for children [12], parents were asked to separately report weekday and weekend screen time. Parents were provided five options, including ‘less than 1 h’, ‘1 to less than 2 h’, ‘2 to less than 3 h’, ‘3 to less than 4 h’ or ‘4 or more hours’. 

#### 2.2.2. Demographic Data

The demographic characteristics included gender (boy, girl or self-identify) and SES based on the neighborhood-level median family Income, which was determined by linking the participants’ home postal code to the median family income data for the corresponding dissemination areas (DA) from the 2016 Census of Canada. Utilizing postal codes and census data has become a common practice for identifying health behaviors and the determinants of health at a population level in Canada [21]. DAs are the smallest geographic units used by Statistics Canada to systematize socio-economic data, and they commonly act as a proxy for neighborhoods [22]. As DAs represent small, homogenous neighborhoods in urban areas, the census data provided a neighborhood-level measure for household income that illustrates the influence of neighborhood income on children’s health behaviors [23]. The SES groups were categorized as low (i.e., < 70,000 CAD), middle (i.e., 70,000–94,999 CAD) and upper-middle/upper income (i.e., > 95,000 CAD). The cut-offs for the income groups were determined by dividing all the DAs in London, Ontario (*n* = 570) into tertiles based on the area’s median family income. 

#### 2.2.3. COVID-19 Constraints

Parents were asked about their family’s approach to COVID-19 safety. First, parents were asked about their child’s current recreational facility use (i.e., indoor and outdoor facilities, only outdoor facilities, no public facilities, or not allowed to use public facilities until vaccinated). As two of the options focused on the restricted use of public recreational facilities, ‘no public facilities’ and ‘not allowed to use public facilities until vaccinated’ were combined, which resulted in three categories for analysis: indoor and outdoor facilities, outdoor facilities only, and no public facilities. Subsequently, parents were asked about their child’s social interaction, specifically whom their child was allowed to be physically active with (i.e., anyone, anyone with physical distancing, or those within the child’s bubble). 

### 2.3. Statistical Analysis

Data from the two surveys were matched and analyzed using SPSS (Version 27; IBM, Armonk, NY, USA). The median and interquartile range (IQR) was calculated to summarize skewed continuous variables or ordinal data. Nominal variables were described using percentages. As the physical activity data were not normally distributed and screen time was a categorical variable, non-parametric tests were performed. A series of Wilcoxon signed-rank tests were used to measure the change in children’s physical activity and screen time (weekday and weekend) from before to during COVID-19. The tests were then repeated for the sub-group analyses (gender, SES, facility use and social interaction). Cohen’s d was calculated to determine the effect size, and they were interpreted as small (*d* = 0.20), medium (*d* = 0.50), and large (*d* = 0.80) based on the criteria proposed by Cohen [24]. A *p*-value ≤ 0.05 was considered statistically significant.

## 3. Results

### 3.1. Sample Characteristics

A total of 1902 grade six families were invited to participate in the G5AP study. Of those contacted, 719 parents (37.8%) completed the before COVID-19 survey and 231 parents (12.2%) completed the COVID-19 survey. Once the datasets were paired, 98 parents provided survey data before and during COVID-19. Three participants were excluded due to incomplete data (i.e., parents completing less than 50% of the survey), resulting in 95 parent responses included in the study. Sample characteristics are displayed in Table 1.

### 3.2. Overall Changes in Physical Activity and Screen Time

The Wilcoxon signed-ranks test indicated a statistically significant decrease in parent-reported children’s physical activity from 4.39 (SD = 1.96) days per week before COVID-19 to 3.78 (SD = 2.19) days per week during the COVID-19 pandemic (Z = −2.53, *p* = 0.01, *d* = 0.18; Table 2). Parents also reported that children engaged in an additional hour per day of recreational screen time on weekdays (Z = −4.61, *p* < 0.01, *d* = 0.33) and weekends (Z = −3.79, *p* < 0.01, *d* = 0.27).

### 3.3. Sub-Group Analyses

In terms of gender, girls experienced a larger decline in physical activity during COVID-19 (Z = −2.80, *p* < 0.01, *d* = 0.20), while boys displayed minor declines in physical activity (Z = −1.05, *p* = 0.29) days per week (Table 3). Recreational screen time significantly increased for boys and girls during COVID-19, with boys having a greater increase in weekday screen time (Z = −3.74, *p* < 0.01, *d* = 0.27) compared to girls (Z = −2.76, *p* < 0.01, *d* = 0.20), and both genders having an hour increase on weekends.

There were significant declines in physical activity for upper/upper-middle income children (Z = −3.26, *p* < 0.01, *d* = 0.24), while low (Z = −0.10, *p* = 0.92) and middle income children (Z = −0.41, *p* = 0.68) did not have significant changes in physical activity (Table 4). Weekday recreational screen time significantly increased for middle income (Z = −3.21, *p* < 0.01, *d* = 0.23) and upper/upper-middle children (Z = −3.26, *p* = 0.01, *d* = 0.24). Weekend recreational screen time increased for all groups, with upper/upper-middle income children (Z = −3.24, *p* < 0.01, *d* = 0.24) having statistically significant increases. 

With respect to COVID-19 constraints, children who had access to indoor and outdoor facilities displayed the greatest decrease in their physical activity (Z = −2.37, *p =* 0.02, *d* = 0.17), and the greatest increase in weekday recreational screen time (Z = −3.89, *p* < 0.01, *d* = 0.28) and weekend recreational screen time (Z = −3.71, *p* < 0.01, *d* = 0.27; Table 5). Additionally, children who were not allowed to use public physical activity facilities had a significant increase in weekday screen time (Z = −2.42, *p =* 0.02, *d*= 0.18), but there were no changes to their physical activity or weekend screen time. Children who only had access to outdoor recreational facilities had minimal changes to their physical activity or screen time behaviors. Conversely, all of the social interaction groups (i.e., all friends and family, all friends and family with physical distancing, and those within the child’s ‘bubble’) had significant increases in weekday and weekend screen time but did not experience a significant change in physical activity (Table 6).

## 4. Discussion

This study examined how children’s physical activity and screen time have changed during the COVID-19 pandemic. The study also assessed if the changes in these health behaviors differed between subgroups of children, including gender, SES, and family COVID-19 practices. Overall, parents reported declines in children’s physical activity and additional weekday and weekend recreational screen time. These findings align with other research on children’s physical activity and screen time during the COVID-19 pandemic [25,26,27,28,29,30,31,32]. For example, Moore et al. [26] conducted a study with a national sample of Canadian children during the early stages of COVID-19. When asked to compare their child’s current health behaviors to before COVID-19, parents reported that their child had immediate declines in all forms of physical activity and an increase in recreational screen time, primarily social media use [26]. In the United States, a nationally distributed parent-reported survey found immediate declines in children’s physical activity during COVID-19 lockdowns and greater engagement in sedentary activities [32]. Similarly, a longitudinal study that collected both self-reported and accelerometer data from children ages 4 to 18 years in the Netherlands indicated that daily physical activity levels declined and sedentary time increased during the pandemic [28]. 

One explanation for these health behavior changes is that COVID-19 has intensified or introduced barriers to physical activity participation. Parents have identified opportunities outside of the house, such as physical education classes, recess time, and extracurricular activities, as important sources of physical activity for their children [17,33]; however, these opportunities have not been available or limited during the COVID-19 pandemic. In the Canadian context, organized sports and activities are the predominant form of physical activity for children, with only 21% of children engaging in unstructured physical activity [10]. Access to available recreational programming has been a challenge because of public health protocols restricting the number of in-person activities, mandating smaller class sizes and converting community centers into vaccination centers, thus limiting the number of local recreational opportunities for children. Organized activities are also financially inaccessible to some families due to higher job instability created by layoffs and downsizing of businesses. As a result, parents may worry about affording their mortgage or rent, bills, and necessities, and are unable to prioritize physical activity programs [34]. 

Children being confined to their homes has also placed additional pressure on parents to facilitate healthy lifestyle behaviors. Moore et al. [26] reported that parents’ ability to support physical activity and discourage screen-based activities facilitated movement behaviors during the COVID-19 pandemic. Similarly, parents’ perceived ability to limit children’s screen time is an important factor in meeting screen time and physical activity recommendations [35]. As children are spending more time at home, screen-based activities have become a convenient, accessible, and cost-effective option [36]. Some parents describe screens as necessary, because they engage their children in an activity while they are working, and provide children with an opportunity to socialize with friends and family members they cannot see due to gathering restrictions [37]. The combination of these factors created by COVID-19 can restrict the number of recreational opportunities available to children and encourage screen use, resulting in unhealthy lifestyle behaviors. 

The sub-group analyses identified demographic differences in physical activity and screen time during the pandemic. Girls had greater changes in physical activity and weekend screen time compared to boys; however, boys had greater increases in weekday screen time than girls. Previous studies have shown that boys are more likely to meet the physical activity recommendations than girls [26,35]. However, our findings contradict these studies, with girls and boys having similar physical activity levels during COVID-19. This discrepancy may be due to the sample of girls included in the study being more active than the national average, as girls having higher parent-reported physical activity levels from the before COVID-19 survey are contrary to the literature on children’s physical activity [10,13,38]. 

Additionally, children from higher SES households had significant declines in physical activity and significant increases in screen time. Children in higher-income households tend to have a greater number of sport equipment options at home [39]; nonetheless, they are more likely to be enrolled in organized sports, and the declines in physical activity may be due to the limited availability of these activities [40]. Lower physical activity levels may also be attributed to low levels of independent mobility (i.e., the freedom to travel around the neighborhood without parental supervision), as parents may be unable to supervise children outside of the house and are uncomfortable with allowing the child to play without supervision, limiting participation in unstructured activities and resulting in increased screen time [41]. Children from low-SES households did not experience this decline in physical activity and it may be attributed to children’s lower parent-reported physical activity levels and higher screen time, and their lower enrollment rates in organized activities prior to the COVID-19 pandemic [40]. 

In terms of COVID-19 constraints, the findings indicated that children who were only provided access to outdoor facilities were able to maintain their physical activity levels, while those who had permission to use all recreational facilities had significant changes in physical activity and screen time. This aligns with other studies that showed children who took part in greater amounts of outdoor physical activity (e.g., walking or biking) were more likely to meet the Canadian movement guidelines [35]. There could be an argument that children with access to all facilities should be able to maintain their physical activity compared to those who are restricted, since the former have a greater variety of options [42]. Yet, children who had access to all facilities may primarily use indoor places, which are less or no longer accessible during the pandemic. Kelso et al. [42] identified that home and indoor recreational places were the locations that children get the greatest daily MVPA, while home and school were the places with the highest sedentary time. The restricted use of indoor facilities and additional time at home may result in lower physical activity levels and higher screen time, as children could be disinterested in the activity options available in the house [32]. Alternatively, all social interaction groups had a significant effect on children’s screen time, but did not experience significant changes in their physical activity. This finding suggests that restrictions on socialization did not considerably impact the physical activity levels of elementary school-aged children, and the ability to engage in activities with either a peer or family member supported children’s physical activity during the pandemic. Research has shown that engaging in activities with peers [43,44] and family members [45,46] results in higher physical activity levels amongst children. Potentially, other outcomes from the COVID-19 protocols had a stronger effect on children’s movement behaviors. 

It is evident that additional public health measures that provide children with physical activity and non-screen-based activities are needed through this pandemic. The current public health measures in place are preventing transmission of the virus, but they are also creating barriers to important services. To support families through the pandemic, parents need resources for non-screen activities that can increase their child’s physical activity that consider the unique challenges created by COVID-19 [17]. For instance, activities in outdoor spaces, such as parks, playgrounds and trails, can give children the opportunity to be active with friends and family with adequate space for physical distancing. Physical activity in outdoor spaces has also been associated with various health benefits for children, such as improved social competence [47] and mental health [48]. In addition, parents should encourage unstructured activities after school and on weekends. Unstructured activities, such as walking, biking and playground games, can replace the unavailable organized activities during the pandemic. Additional opportunities for children to engage in imaginative play can also have positive impacts on children’s creativity, cognitive learning, and social and emotional development [49]. 

To our knowledge, this is the first longitudinal study examining the physical activity and screen time changes amongst Canadian children, with baseline data collected prior to the COVID-19 pandemic. The use of longitudinal data allowed the research team to examine the same children’s physical activity and screen time at two time points, providing a strong representation of the changes to children’s health behaviors, and minimizing recall bias. Additionally, this study contributes to the understanding of how the COVID-19 public health protocols implemented in Canada have influenced children’s physical activity and screen time. Despite these strengths, study limitations must be acknowledged. For instance, this study was conducted with 95 parents from a convenience sample of previous G5AP participants; however, a comparison of the study’s sample to the London population indicates that characteristics of the sample (i.e., family income, immigration status, visible minority status) are similar to that of the general population. Parent-reported surveys also introduce the potential for volunteer (e.g., parents may prioritize physical activity for their children) or desirability bias (e.g., misrepresent their children’s movement behaviors), which may have overestimated the physical activity data and underestimated screen time. Additionally, differences in regional and national COVID-19 policies may have distinct influences on physical activity levels and screen time behaviors, impacting the generalizability of the findings to other communities. The applicability of the findings are also limited to elementary school-aged children, as this study collected data from the parents of children ages 10 to 12 years; consequently, the findings may not reflect the changes in the movement behaviors of primary school (ages 5–8 years) or high school-aged children (ages 13–17 years) during the COVID-19 pandemic. Seasonality also needs to be considered. In colder months, physical activity levels tend to decrease, while sedentary activities (i.e., screen time) tend to increase [50]. As the COVID-19 survey was distributed from November to December 2020, while the before COVID-19 survey was completed from May 2019 to February 2020, seasonality may influence the responses if parents completed the surveys at different times of the year.

## 5. Conclusions

The findings from this study provide context into how the COVID-19 protocols have negatively influenced children’s movement behaviors as children are engaging in less physical activity and more screen-based activities during the COVID-19 pandemic. These findings exemplify the need for resources that can engage children in greater amounts of physical activity and reduce screen time, and to prioritize outdoor play for this group. Resources also need to be equitable to ensure they can positively impact the movement behaviors of different genders and SES groups and can be adapted based on a family’s COVID-19 constraints. Moving forward, additional studies are needed to assess the recreational programming options available to children, identify the activities children are missing, and highlight protocols that need to be in place to facilitate children’s return to physical activity in public spaces.

## Figures and Tables

**Table 1:** Characteristics of the sample population (*n* = 95).

Characteristic		*n* (%)
Demographics		
Gender	Boy	48 (50.5)
	Girl	47 (49.5)
	Self-identify	0 (0)
Age	10 years old	15
	11 years old	79
	12 years old	1
Ethnicity	White/Caucasian	57 (60.0)
	Visible Minority	31 (32.6)
	Mixed Race	7 (7.4)
Years in Canada	Born in Canada	69 (72.6)
	More than 5 years	8 (8.4)
	3 to 4 years	8 (8.4)
	1 to 2 years	9 (9.5)
	Less than 1 year	1 (1.1)
Household Size	2 people	5 (5.3)
	3 people	13 (13.7)
	4 people	42 (44.2)
	5 people	30 (31.6)
	6 people	5 (5.3)
MFI (CAD $)	Low	23 (24.2)
	Middle	22 (23.2)
	High	50 (52.6)
**COVID-19** **Constraints**		
School Model	In-person learning	83 (87.4)
	Remote learning	11 (11.6)
	Home schooling	1 (1.1)
Facility Use	Indoor and outdoor facilities	49 (51.6)
	Only outdoor facilities	21 (22.1)
	No indoor or outdoor facilities	25 (26.3)
Social Interaction	All friends and family	28 (29.5)
	Friends and family with physical distancing	38 (40.0)
	Those within the child’s bubble	29 (30.5)

MFI = Median Family Income.

**Table 2:** Changes in children’s physical activity and screen time from before to during COVID-19 (*n* = 95).

**Health Behavior**	**Before COVID-19**		**During COVID-19**		**−ve Rank** **(*n*)**	**+ve Rank** (*n*)	**Z ^a^**	***p***	***d***
	$\bar{x}$ **(SD)**	**Median (IQR)**	$\bar{x}$ **(SD)**	**Median (IQR)**					
Physical activity (days/week)	4.39 (1.96)	4 (3–6)	3.78 (2.19)	4 (2–5)	46	23	−2.53	0.01	0.18
Weekday recreational screen time (h/day)	2.12 (0.94)	1–2 (0–3)	2.63 (1.08)	2–3 (1–3)	10	47	−4.61	<0.01	0.33
Weekend recreational screen time (h/day)	3.34 (1.13)	2–3 (1–4)	3.83 (1.17)	3–4 (2–4+)	14	46	−3.79	<0.01	0.27

$\bar{x}$= mean; SD = standard deviation; IQR = interquartile range; *d* = Cohen’s d effect size; a = based on positive ranks. Physical activity is the number of days per week a child accumulated 60 min of moderate-to-vigorous physical activity (MVPA). Screen time is the number of hours a child uses a screen-based device in a day, and parents were asked to select one of the five options: ‘less than 1 h’, ‘1 to less than 2 h’, ‘2 to less than 3 h’, ‘3 to less than 4 h’ or ‘4 or more hours’.

**Table 3:** Gender differences in children’s physical activity and screen time from before to during COVID-19.

**Boys (*n* = 48)**									
**Health Behavior**	**Before COVID-19**		**During COVID-19**		**−ve Rank** **(*n*)**	**+ve Rank** **(*n*)**	**Z ^a^**	***p***	***d***
	$\bar{x}$ **(SD)**	**Median (IQR)**	$\bar{x}$ **(SD)**	**Median (IQR)**					
Physical activity (day/week)	4.21 (2.07)	4 (3–6)	3.77 (2.28)	4 (2–5.75)	22	15	−1.05	0.29	0.08
Weekday recreational screen time (h/day)	2.21 (0.93)	1–2 (1–3)	2.79 (1.03)	2–3 (1–3.75)	5	28	−3.74	<0.01	0.27
Weekend recreational screen time (h/day)	3.51 (1.10)	2–3 (2–4+)	3.98 (1.06)	3–4 (2–4+)	8	21	−2.35	0.02	0.17
**Girls (*n* = 47)**									
**Health Behavior**	**Before COVID-19**		**During COVID-19**		**−ve Rank** **(*n*)**	**+ve Rank** **(*n*)**	**Z ^a^**	***p***	***d***
	$\bar{x}$ **(SD)**	**Median (IQR)**	$\bar{x}$ (**SD**)	**Median (IQR)**					
Physical activity (day/week)	4.57 (1.87)	5 (3–6)	3.79 (2.13)	4 (2–5)	24	8	−2.80	<0.01	0.20
Weekday recreational screen time (h/day)	2.02 (0.94)	1–2 (0–3)	2.47 (1.12)	1–2 (1–3)	5	19	−2.76	<0.01	0.20
Weekend recreational screen time (h/day)	3.17 (1.15)	2–3 (1–4)	3.68 (1.27)	3–4 (2–4+)	6	25	−3.19	<0.01	0.23

$\bar{x}$ = mean; SD = standard deviation; IQR = interquartile range; *d* = Cohen’s d effect size; a = based on positive ranks. Physical activity is the number of days per week a child accumulated 60 min of moderate-to-vigorous physical activity (MVPA). Screen time is the number of hours a child uses a screen-based device in a day, and parents were asked to select one of the five options: ‘less than 1 h’, ‘1 to less than 2 h’, ‘2 to less than 3 h’, ‘3 to less than 4 h’ or ‘4 or more hours’.

**Table 4:** Median family income differences in children’s physical activity and screen time from before to during COVID-19.

**Low (*n* = 23)**									
**Health Behavior**	**Before COVID-19**		**During COVID-19**		**−ve Rank** **(*n*)**	**+ve Rank** **(*n*)**	**Z ^a^**	***p***	***d***
	$\bar{x}$ **(SD)**	**Median (IQR)**	$\bar{x}$ **(SD)**	**Median (IQR)**					
Physical activity (day/week)	3.39 (1.75)	3 (2–5)	3.35 (2.25)	3 (2–5)	9	8	−0.10	0.92	0.01
Weekday recreational screen time (h/day)	2.48 (0.99)	1–2 (1–3)	2.70 (1.19)	2–3 (1–4)	5	8	−1.17	0.24	0.08
Weekend recreational screen time (h/day)	3.70 (1.11)	3–4 (2–4+)	4.04 (1.19)	4+ (2–4+)	4	11	−1.28	0.20	0.09
**Middle (*n* = 22)**									
**Health Behavior**	**Before COVID-19**		**During COVID-19**		**−ve Rank** **(*n*)**	**+ve Rank** **(*n*)**	**Z ^a^**	***p***	***d***
	$\bar{x}$ **(SD)**	**Median (IQR)**	$\bar{x}$ **(SD)**	**Median (IQR)**					
Physical activity (day/week)	3.95 (2.06)	4 (3–5)	3.59 (2.11)	3.5 (2–5)	10	7	−0.41	0.68	0.03
Weekday recreational screen time (h/day)	1.91 (0.92)	1–2 (0–3)	2.82 (1.10)	2–3 (1–3.25)	1	14	−3.21	<0.01	0.23
Weekend recreational screen time (h/day)	3.32 (1.32)	2–3 (1–4+)	3.82 (1.19)	3–4 (2–4+)	4	11	−1.78	0.08	0.13
**Upper/Upper-Middle (*n* = 50)**									
**Health Behavior**	**Before COVID-19**		**During COVID-19**		**−ve Rank** **(*n*)**	**+ve Rank ** **(*n*)**	**Z ^a^**	***p***	***d***
	$\bar{x}$ **(SD)**	**Median (IQR)**	$\bar{x}$ **(SD)**	**Median (IQR)**					
Physical activity (day/week)	5.04 (1.82)	5 (4–7)	4.06 (2.21)	4 (2–6)	27	8	−3.26	<0.01	0.24
Weekday recreational screen time (h/day)	2.04 (0.89)	1–2 (0–3)	2.52 (1.04)	1–2 (1–3)	4	25	−3.26	0.01	0.24
Weekend recreational screen time (h/day)	3.18 (1.03)	2–3 (1–4)	3.74 (1.18)	3–4 (2–4+)	6	24	−3.24	<0.01	0.24

$\bar{x}$= mean; SD; standard deviation; IQR = interquartile range; *d* = Cohen’s d effect size; a = based on positive ranks. Physical activity is the number of days per week a child accumulated 60 min of moderate-to-vigorous physical activity (MVPA). Screen time is the number of hours a child uses a screen-based device in a day, and parents were asked to select one of the five options: ‘less than 1 h’, ‘1 to less than 2 h’, ‘2 to less than 3 h’, ‘3 to less than 4 h’ or ‘4 or more hours’.

**Table 5:** Facility use differences in children’s physical activity and screen time from before to during COVID-19.

**Indoor and Outdoor Facilities (*n* = 49)**									
**Health Behavior**	**Before COVID-19**		**During COVID-19**		**−ve Rank** **(*n*)**	**+ve Rank** **(*n*)**	**Z ^a^**	***p***	***d***
	$\bar{x}$ **(SD)**	**Median (IQR)**	$\bar{x}$ **(SD)**	**Median (IQR)**					
Physical activity (day/week)	4.82 (1.93)	5 (3–7)	4.02 (2.12)	4 (2–5.50)	26	10	−2.37	0.02	0.17
Weekday recreational screen time (h/day)	2.06 (0.91)	1–2 (0.5–2)	2.65 (1.09)	2–3 (1–3)	4	28	−3.89	<0.01	0.28
Weekend recreational screen time (h/day)	3.29 (1.11)	2–3 (1.50–4)	3.92 (1.10)	3–4 (2–4+)	5	27	−3.71	<0.01	0.27
**Only Outdoor Facilities (*n* = 21)**									
**Health Behavior**	**Before COVID-19**		**During COVID-19**		**−ve Rank** **(*n*)**	**+ve Rank ** **(*n*)**	**Z ^a^**	***p***	***d***
	$\bar{x}$ **(SD)**	**Median (IQR)**	$\bar{x}$ **(SD)**	**Median (IQR)**					
Physical activity (day/week)	3.90 (2.12)	3 (3–5.50)	3.81 (2.21)	3 (2–6.50)	7	5	−0.28	0.78	0.02
Weekday recreational screen time (h/day)	2.19 (1.03)	1–2 (0–3)	2.38 (1.12)	1–2 (1–3)	3	6	−1.12	0.25	0.08
Weekend recreational screen time (h/day)	3.24 (1.18)	2–3 (1–4)	3.43 (1.36)	3–4 (1.50–3.50)	4	8	−0.99	0.32	0.07
**No Indoor or Outdoor Facilities (*n* = 25)**									
**Health Behavior**	**Before COVID-19**		**During COVID-19**		**−ve Rank** **(*n*)**	**+ve Rank ** **(*n*)**	**Z ^a^**	***p***	***d***
	$\bar{x}$ **(SD)**	**Median** **(IQR)**	$\bar{x}$ **(SD)**	**Median (IQR)**					
Physical activity (day/week)	3.96 (1.81)	4 (2.50–5)	3.28 (2.34)	3 (1–5)	13	8	−1.24	0.21	0.09
Weekday recreational screen time (h/day)	2.16 (0.94)	1–2 (0–3)	2.80 (1.04)	2–3 (1–4)	3	13	−2.42	0.02	0.18
Weekend recreational screen time (h/day)	3.52 (1.16)	3–4 (2–3.50)	4.00 (1.12)	3–4 (2–4+)	5	11	−1.45	0.15	0.11

$\bar{x}$= mean; SD; standard deviation; IQR = interquartile range; *d* = Cohen’s d effect size; a = based on positive ranks. Physical activity is the number of days per week a child accumulated 60 min of moderate-to-vigorous physical activity (MVPA). Screen time is the number of hours a child uses a screen-based device in a day, and parents were asked to select one of the five options: ‘less than 1 h’, ‘1 to less than 2 h’, ‘2 to less than 3 h’, ‘3 to less than 4 h’ or ‘4 or more hours’.

**Table 6:** Social interaction differences in children’s physical activity and screen time from before to during COVID-19.

**All Friends and Family (*n* = 28)**									
**Health Behavior**	**Before COVID-19**		**During COVID-19**		**−ve Rank** **(*n*)**	**+ve Rank** **(*n*)**	**Z ^a^**	***p***	***d***
	$\bar{x}$ **(SD)**	**Median (IQR)**	$\bar{x}$ **(SD)**	**Median (IQR)**					
Physical activity (day/week)	4.75 (2.14)	5 (3–7)	4.21 (2.17)	5 (3–6)	14	6	−1.27	0.21	0.09
Weekday recreational screen time (h/day)	2.43 (1.03)	1–2 (1–3)	3.00 (1.28)	2–3 (1–4)	3	15	−2.91	<0.01	0.21
Weekend recreational screen time (h/day)	3.75 (1.24)	3–4 (1.50–4+)	4.18 (1.22)	4+ (3–4+)	4	14	−2.10	0.04	0.15
**All Friends and Family with Physical Distancing (*n* = 38)**									
**Health Behavior**	**Before COVID-19**		**During COVID-19**		**−ve Rank** **(*n*)**	**+ve Rank ** **(*n*)**	**Z ^a^**	***p***	***d***
	$\bar{x}$ **(SD)**	**Median (IQR)**	$\bar{x}$ **(SD)**	**Median (IQR)**					
Physical activity (day/week)	4.61 (1.90)	5 (3–6)	4.16 (1.97)	4 (2–6)	18	8	−1.51	0.13	0.11
Weekday recreational screen time (h/day)	1.89 (0.86)	1–2 (0–2)	2.39 (1.03)	1–2 (1–3)	3	19	−3.05	<0.01	0.22
Weekend recreational screen time (h/day)	2.97 (0.92)	2–3 (1–3)	3.45 (1.16)	2.50 (2–4)	5	18	−2.35	0.02	0.17
**Those within the Child’s ‘Bubble’ (*n* = 29)**									
**Health Behavior**	**Before COVID-19**		**During COVID-19**		**−ve Rank** **(*n*)**	**+ve Rank ** **(*n*)**	**Z ^a^**	***p***	***d***
	$\bar{x}$ **(SD)**	**Median** **(IQR)**	$\bar{x}$ **(SD)**	**Median (IQR)**					
Physical activity (day/week)	3.76 (1.83)	4 (2.50–5)	2.86 (2.30)	3 (1–5)	14	9	−1.81	0.07	0.13
Weekday recreational screen time (h/day)	2.11 (0.88)	1–2 (0–3)	2.59 (0.87)	2–3 (1–3)	4	13	−2.05	0.04	0.15
Weekend recreational screen time (h/day)	3.43 (1.17)	3–4 (2–4)	4.00 (1.04)	3–4 (2–4+)	5	14	−2.16	0.03	0.16

$\bar{x}$= mean; SD; standard deviation; IQR = interquartile range; *d* = Cohen’s d effect size; a = based on positive ranks. Physical activity is the number of days per week a child accumulated 60 min of moderate-to-vigorous physical activity (MVPA). Screen time is the number of hours a child uses a screen-based device in a day, and parents were asked to select one of the five options: ‘less than 1 h’, ‘1 to less than 2 h’, ‘2 to less than 3 h’, ‘3 to less than 4 h’ or ‘4 or more hours’.

## Data Availability

Due to research ethics board requirements, data are not available for distribution.
